# Viral elimination is essential for improving surgical outcomes of hepatitis C virus‐related hepatocellular carcinoma: Multicenter retrospective analysis

**DOI:** 10.1002/ags3.12377

**Published:** 2020-07-23

**Authors:** Masao Nakajima, Shogo Kobayashi, Hiroshi Wada, Akira Tomokuni, Hidenori Takahashi, Takehiro Noda, Hiroto Matsui, Satoshi Matsukuma, Shinsuke Kanekiyo, Yoshitaro Shindo, Yukio Tokumitsu, Yuki Nakagami, Nobuaki Suzuki, Shigeru Takeda, Masahiro Tanabe, Katsuyoshi Ito, Yoshinobu Hoshii, Hidetoshi Eguchi, Hiroaki Nagano

**Affiliations:** ^1^ Department of Gastroenterological, Breast and Endocrine Surgery Yamaguchi University Graduate School of Medicine Yamaguchi Japan; ^2^ Department of Gastroenterological Surgery Graduate School of Medicine Osaka University Osaka Japan; ^3^ Department of Surgery Osaka International Cancer Institute Osaka Japan; ^4^ Department of Gastroenterological surgery Osaka General Medical Center Osaka Japan; ^5^ Department of Radiology Yamaguchi University Graduate School of Medicine Yamaguchi Japan; ^6^ Department of Diagnostic pathology Yamaguchi University Hospital Yamaguchi Japan

**Keywords:** hepatectomy, hepatitis C virus, hepatocellular carcinoma, regression of liver fibrosis, sustained virologic response

## Abstract

**Aim:**

The impact of sustained virologic response (SVR) on surgical outcomes for patients with hepatitis C virus (HCV)‐related hepatocellular carcinoma (HCC) remains controversial. This study aimed to evaluate the influence of SVR on long‐term surgical outcomes after hepatectomy.

**Methods:**

This multicenter study included 504 patients who underwent curative resection for HCV‐related HCC. Patients with a history of HCC treatment, HBV infection, poor liver function, and tumor with major vascular invasion were excluded. Long‐term surgical outcomes (overall survival [OS] and recurrence‐free survival [RFS]) among patients who achieved SVR before hepatectomy (Pre‐SVR group: 58 patients), after hepatectomy (Post‐SVR group: 54 patients), and without SVR (Non‐SVR group: 186 patients) were compared after adjusting for 13 confounding factors. Using the surgically resected specimens, comparison of the pathological changes in liver fibrosis between the first and second hepatectomy were analyzed.

**Results:**

Patients with SVR were younger, had better liver function, and less liver fibrosis compared to patients without SVR. Propensity score‐matched OS and RFS were significantly better in Pre‐SVR group than Non‐SVR group (*P* = .029 and *P* = .009, respectively). Inverse probability‐weighted OS and RFS were also significantly better in the Post‐SVR group (*P* = .001 and *P* = .021, respectively) than in the Non‐SVR group. Histopathological evaluation revealed that only the patients with SVR had regression of liver fibrosis (*P* < .05).

**Conclusion:**

Achievement of SVR before or after hepatectomy is essential for improving long‐term surgical outcomes in patients with HCV‐related HCC.

## Graphical abstract

Although the achievement of sustained virologic response (SVR) is essential for improving survival in patients with hepatitis C virus (HCV) infection, its importance on surgical outcomes for HCV‐related hepatocellular carcinoma (HCC) is still controversial. In this multicenter study, we demonstrated the importance of achieving SVR before or after hepatectomy for improving long‐term surgical outcomes. These results can encourage patients with HCV‐related HCC to undergo antiviral therapy regardless of the timing.

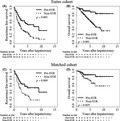

## 1 INTRODUCTION

Hepatocellular carcinoma (HCC) is one of the leading causes of cancer mortality worldwide.^1^ Among the several risk factors of HCC, chronic hepatitis C virus (HCV) infection is a vital cause of HCC. Elimination of HCV and achievement of sustained virologic response (SVR) by interferon (IFN) therapy prevents or delays the progression of fibrosis/cirrhosis, decompensation, and the incidence of primary HCC, leading to improved survival.^2^ However, it is well‐known that HCC sometimes develops even after achieving SVR, with a yearly reported incidence of 0.8%‐1.5%, depending on the extent of liver fibrosis.^3^ To improve the survival of these patients, curative treatment for HCC is essential.

Previous reports have demonstrated the possible favorable effect of SVR before hepatectomy on surgical outcomes^4^, ^5^, ^6^, ^7^, ^8^, ^9^, ^10^; however, the true impact of SVR is not well‐documented for various reasons. Furthermore, not all patients with HCV‐related HCC can achieve SVR before hepatectomy; some achieve it after. However, few reports are available about the impact of postoperative SVR on surgical outcomes.^11^, ^12^, ^13^ Therefore, it remains unclear whether patients with curative resection of HCC should receive antiviral therapy or not.

As reported, long‐term surgical outcomes of HCC are influenced by several factors. These include tumor characteristics (size, number, macroscopic findings of the tumor, extent of vascular invasion), background liver function, and fibrosis.^13^, ^14^ Therefore, one of the vital reasons why the true impact of SVR before or after hepatectomy is still being debated could be due to small sample sizes of patients compared to the several confounding factors that need to be adjusted for, and which influence the surgical outcomes.

In this retrospective multicenter analysis, the impact of SVR before and after hepatectomy on the long‐term surgical outcomes of HCV‐related HCC was investigated after statistically minimizing bias arising from patients' background characteristics. The relationship between achieving SVR and change in liver fibrosis was also investigated in this study.

## 2 PATIENTS AND METHOD

### 2.1 Study design and patients

This was a multicenter (Department of Surgery, Osaka University; Department of Surgery, Osaka International Cancer Institute; and Department of Gastroenterological, Breast, and Endocrine Surgery, Yamaguchi University) retrospective observational study. The study enrolled 504 chronically HCV‐infected patients that underwent curative resection for HCC between 1996 and 2015. Being chronically HCV‐infected was defined as positive HCV antibody at HCC diagnosis. Among the 504 patients, 128, nine, 16, and 57 with a history of HCC treatment,^15^ hepatitis‐B virus infection (defined as serological hepatitis‐B virus surface antigen positive),^16^ major vascular invasion (i.e. with Vp3 or higher and Vv2 or higher),^17^ and poor liver function (Child Pugh B),^18^ respectively, were excluded because these factors strongly influence HCC surgical outcomes (some patients had multiple characteristics). Additionally, 17 patients with observational periods less than one year were also excluded to ensure the accuracy in the analysis. The remaining 298 patients were classified into three groups: achieved SVR before hepatectomy (Pre‐SVR group: N = 58), achieved SVR after hepatectomy (Post‐SVR group: N = 54), and no SVR achievement all the time (Non‐SVR group: N = 186). With regard to the antiviral therapy, all patients in the Pre‐SVR group received antiviral therapy with IFN‐based therapy; on the other hand, 26 patients in the Post‐SVR group received antiviral therapy with direct acting antivirals (DAAs). The median interval from achieving SVR to operation was 80 months in the Pre‐SVR group. The median interval from operation to achieving SVR was 32 months in the Post‐SVR group. Furthermore, to precisely evaluate the impact of SVR after hepatectomy, patients with tumor recurrence before achieving SVR (N = 22) and those with recurrence within 6 months after hepatectomy (Non‐SVR group: N = 20) were excluded because we had started antiviral therapy for the patients without recurrence for at least 6 months after hepatectomy. The SVR group was defined as patients with serum negative HCV‐RNA, 24 weeks after antiviral therapy completion. The non‐SVR group included the 26 patients who did not achieve SVR by preoperative IFN therapy, one patient who did not achieve SVR by postoperative DAA therapy, one patient who did not achieve SVR by pre‐ and postoperative antiviral therapy, and the 158 patients without pre‐ and postoperative antiviral therapy. Based on a 2‐year cut‐off that was generally adopted for classifying HCC recurrence into early and late types, we set the 2 years as the cut‐off between early and late recurrence in this study.^19^ The cumulative recurrence rate in the late phase was evaluated in recurrence‐free patients, 4 years after surgery. This study was approved by the Human Ethics Committee in Yamaguchi University (approval no. H30‐046) and all patients provided written informed consent.

### 2.2 Diagnosis and surgical treatment of HCC

Diagnosis of HCC was performed by dynamic contrast‐enhanced abdominal computed tomography (CT) or gadoxetic acid‐enhanced magnetic resonance (MR) imaging. These techniques enhance the precise detection of HCC and its location in order to prevent intrahepatic metastasis residue during hepatectomy. CT images were acquired with different CT‐scans and protocols depending of the institution and the time of the CT exam. MR images were acquired with 1.5 T or 3.0 T systems including T1‐weighted opposed‐phase and in‐phase imaging, T2‐weighted imaging, unenhanced and dynamic contrast‐enhanced T1‐weighted imaging. For dynamic contrast‐enhanced imaging, arterial, portal venous, and late phases were obtained after bolus injection. Hypervascularity in the arterial phase, washout in the portal venous phase, and hypointensity in the hepatobiliary phase of gadoxetic acid‐enhanced MR imaging were considered diagnostic.^16^ The surgical treatment of HCC was decided based on the liver function, location, and size of the tumor. However, we routinely perform a partial resection of the liver to preserve the remnant volume of the liver as much as possible, for future treatment.^17^ Curative resection is defined as the complete removal of all macroscopically evident tumors detected before the operation, by CT or MR imaging.

### 2.3 Follow‐up of patients

At least every 3‐4 months, patients were followed‐up after hepatic resection. During these regular outpatient visits, the following occurred: physical examination, assessment of tumor markers (alpha‐fetoprotein [AFP] and protein induced by vitamin K absence or antagonists‐II), liver biochemical tests, abdominal ultrasonography, CT and MR imaging, or a combination of some of these modalities, to check for intra‐hepatic and extra‐hepatic recurrence. Recurrence was diagnosed using the same criteria as applied to the diagnosis of HCC. Further treatments with repeated hepatectomy, radiofrequency ablation, transcatheter arterial chemoembolization, or other treatment options followed the diagnosis of recurrence, as indicated based on the remnant liver function and tumor characteristics. In this study, recurrence‐free survival (RFS) was defined as the interval between the initial hepatectomy and the detection of the first recurrence or the last follow‐up examination, while overall survival (OS) was calculated based on the time from initial hepatectomy to death from any cause or last follow‐up. After the initial hepatectomy for HCC, the median follow‐up time for the whole study population was 56.3 (range 3.2‐161.2) months. The median follow‐up time for the Pre‐SVR, Post‐SVR, and non‐SVR groups was 51.2 (range 6.1‐161.2), 68.2 (3.2‐157.4), and 53.6 (3.2‐146.5) months, respectively. No significant difference in follow‐up periods occurred between groups, and no hospital deaths occurred during the period.

### 2.4 Antiviral therapy after hepatectomy

Antiviral therapy was administered to patients who met the following inclusion criteria: (a) detectable serum HCV‐RNA; (b) no recurrence for at least 6 months after curative treatment; (c) age ≤80 years; (d) hemoglobin (Hb) concentration ≥10 g/dL; and (e) platelet count ≥7.5 × 10^4^/μL. Such patients had agreed to undergo the treatment after receiving a full explanation regarding the potential benefits and side effects of the treatment.

### 2.5 Histological evaluation

The resected liver specimens were fixed in 10% formalin buffer and stained with hematoxylin‐eosin and, in part, by Mallory‐Azan stain. Histological evaluation of tumor and non‐cancerous liver parenchyma were performed by one pathologist and one hepatologist who were blinded to each patient's treatment, based on the classification system proposed by Japan Liver Cancer Study Group.^20^ Staging of liver fibrosis was evaluated according to the New Inuyama Classification, defined as F0 (no fibrosis), F1 (fibrous portal expansion), F2 (bridging fibrosis), F3 (bridging fibrosis with architectural distortion), or F4 (cirrhosis).^21^

### 2.6 Statistical analysis

Continuous variables are expressed as medians with interquartile range (IQR). Differences between groups were assessed using the chi‐squared test, Fisher's exact test, or the Mann‐Whitney *U* test. All factors which were considered to be relevant to early HCC recurrence were entered into a multivariate logistic regression analysis to determine the adjusted odds ratios (ORs).^13^, ^14^ RFS and OS rates were determined according to the Kaplan‐Meier method and compared using the log‐rank test. To reduce selection bias between the two groups, we used two statistical maneuvers, i.e. propensity score (PS) matching and inverse probability treatment weighting (IPTW) method, and adjusted with the following 13 variables: age, gender, the size of tumor, and the number of tumors, extent of vascular invasion, macroscopic findings of the tumor, tumor differentiation, serum AFP, albumin level, prothrombin level, platelets count, extent of fibrosis, and surgical procedure.^22^ To compare between Pre‐SVR and Non‐SVR group, PS matching was conducted by matching each patient who had Non‐SVR with a patient who had Pre‐SVR in a 1:1 ratio with a caliper width of 0.2. For comparing Non‐SVR and Post‐SVR, the IPTWs were calculated for maintaining the sample size mathematically and applied for creating a pseudo‐population.^23^ Specifically, the pseudo‐population was created weighting the Post‐SVR group by the reciprocal of their conditional probability of achieving postoperative SVR and the Non‐SVR group by the reciprocal of their probability of not achieving SVR, i.e. *w_i_* = *Z_i_/π_i_* + (1 − *Z_i_*)/(1 − *π_i_*), where *i* is the subject, *Z_i_* = 1 is a subject with Post‐SVR, *Z_i_* = 0 is a subject with Non‐SVR, *π_i_* = *P*(*Z* = 1|***X*** *_i_*) is the conditional probability of Post‐SVR given the observed covariates ***X*** *_i_*. All analyses were performed using R language (version 3.5.2), JMP version 9.0 software package (SAS Institute), and the Mac OS Mojave operating system (Apple Computer). A *P*‐value <.05 was considered statistically significant.

## 3 RESULT

### 3.1 Comparison of clinicopathological factors and surgical outcomes between Pre‐SVR and Non‐SVR group

**FIGURE 1:**
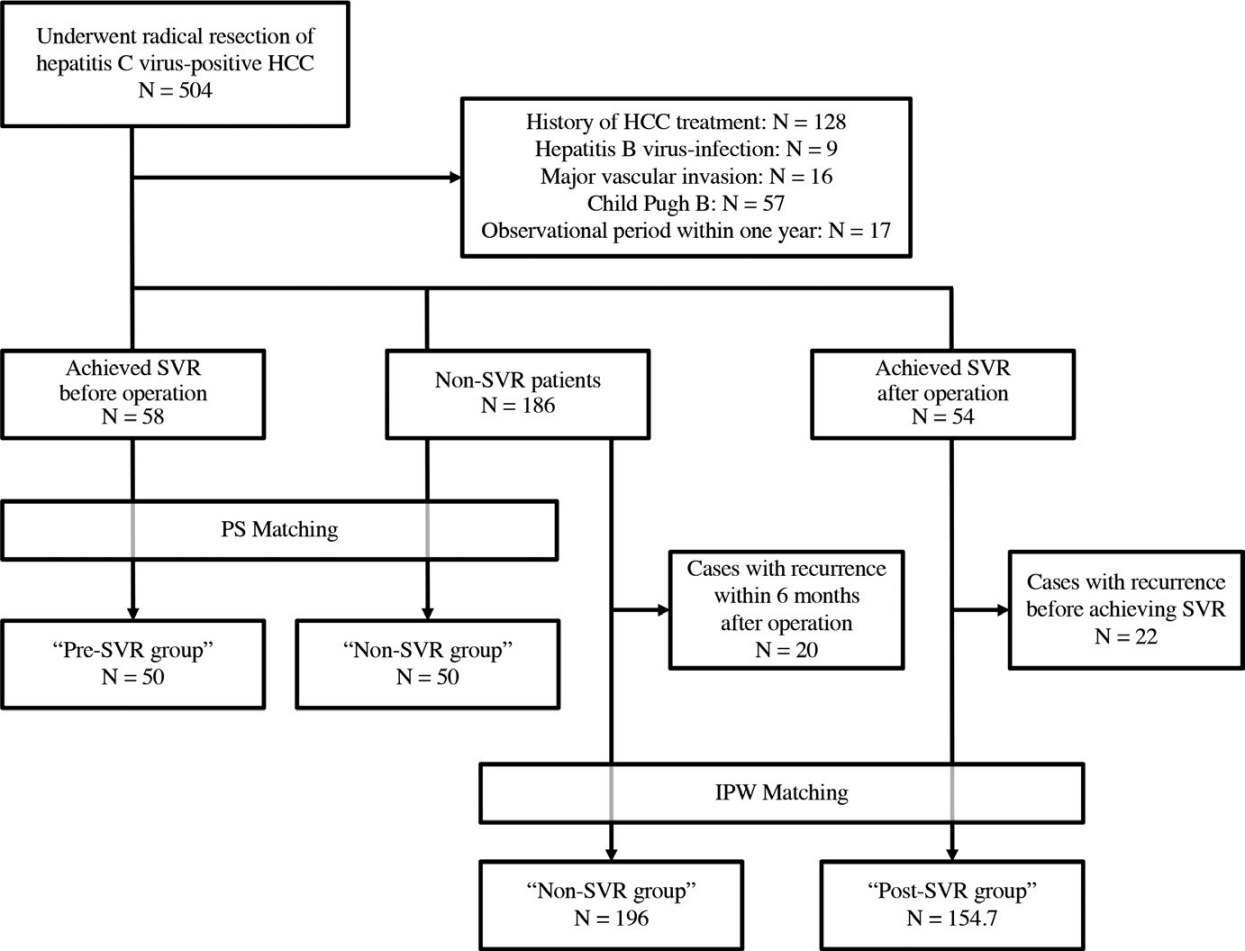
CONSORT diagram of this study. The following 13 baseline variables were used in matching: age, sex, albumin level, prothrombin time, platelet count, the size and number of the tumor, extent of vascular invasion, macroscopic findings of the tumor, tumor differentiation, α‐feto protein level, extent of liver fibrosis and surgical procedure. HCC, hepatocellular carcinoma; IPW, inverse probability weighted; PS, propensity score; SVR, sustained virologic response

The 58 Pre‐SVR patients and the 186 Non‐SVR patients that underwent curative resection for HCC for the first time were analyzed (Figure 1). Patients in the Pre‐SVR group were significantly younger (*P* = .018), had significantly more preserved liver functions, i.e. albumin level (*P* < .001), platelet count (*P* < .001); and significantly lower liver fibrosis (*P* < .001) than those in the Non‐SVR group. For the tumor characteristics, patients in the Pre‐SVR group had significantly lower level of AFP (*P* = .002; Table 1).

**TABLE 1:** Clinicopathological factors of the patients with Non‐SVR and Pre‐SVR

	Unmatched cohort			PS‐matched cohort		
	Non‐SVR	Pre‐SVR	*P* value	Non‐SVR	Pre‐SVR	*P* value
	(N = 186)	(N = 58)		(N = 50)	(N = 50)	
Age (y)	72.0 [66, 77]	69.0 [64, 72.8]	**.018**	70.0 [66, 74.8]	69.0 [63, 72.8]	.168
Male gender, n (%)	134 (72)	46 (79.3)	.354	34 (68)	39 (78)	.368
Liver function						
Platelet (×10^4^/mL)	12.6 [9.6, 15.5]	16.9 [13.7, 20.7]	**<.001**	14.0 [12.3, 19.5]	16.5 [12.9, 19.9]	.224
Albumin > 3.5 g/dL (%)	3.7 [3.5, 4]	4.2 [4, 4.4]	**<.001**	4.1 [3.9, 4.4]	4.1 [3.9, 4.4]	.747
PT (%)	84.0 [77, 92.8]	87.0 [80.0, 92]	.311	84.5 [75, 95]	87.0 [78.2, 92]	.801
Liver fibrosis, n (%)						
F0	2 (1.1)	4 (6.9)	**.001**	2 (4)	3 (6)	.876
F1	19 (10.2)	17 (29.3)		10 (20)	13 (26)	
F2	46 (24.7)	15 (25.9)		14 (28)	14 (28)	
F3	38 (20.4)	7 (12.1)		9 (18)	6 (12)	
F4	79 (42.5)	15 (25.9)		15 (30)	14 (28)	
Tumor factor						
Maximum tumor size (cm)	3.0 [2.2, 4]	3.0 [2, 4]	.424	3.0 [2.2, 3.8]	3.0 [2, 4]	.665
Solitary tumor (%)	140 (75.3)	50 (86.2)	.116	42 (84)	42 (84)	1
Microvascular invasion (%)	57 (30.6)	11 (19.0)	.118	12 (24)	10 (20)	.809
Nodular type (%)	93 (50.0)	31 (53.4)	.758	25 (50)	29 (58)	.547
Tumor differentiation, poor (%)	38 (20.4)	12 (20.7)	1	8 (16)	9 (18)	1
AFP (ng/mL)	18.0 [7, 132]	5.5 [3, 99.8]	**.002**	14.5 [7.0, 71.2]	6.5 [3, 153.5]	.098
Anatomical resection (%)	88 (47.3)	25 (43.1)	.682	20 (40)	20 (40)	1

Notes

Values are medians [interquartile ranges] or numbers (%). Significant *P*‐values are shown in bold.

Abbreviations: AFP, α‐fetoprotein; PS, propensity score; SVR, sustained virological response.

**FIGURE 2:**
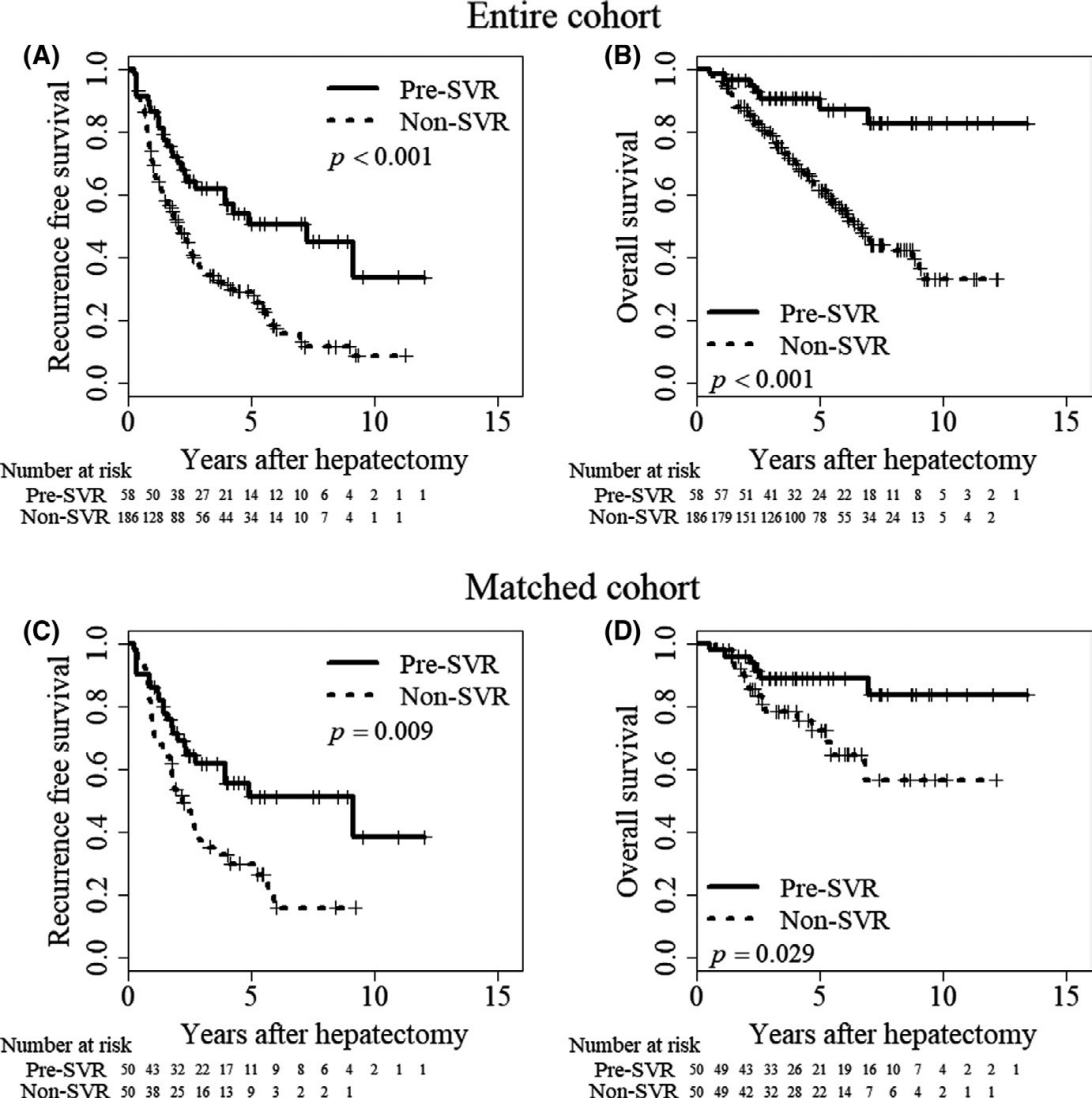
Surgical outcomes of patients in the Non‐SVR group and Pre‐SVR group. A, Recurrence‐free survival (*P* < .001) and (B) overall survival (*P* < .001) in the entire cohort. C, Recurrence‐free survival (*P* = .009) and (D) overall survival (*P* = .029) in the PS‐matched cohort. The Kaplan‐Meier method and log‐rank testing were used to assess the recurrence‐free survival and overall survival rates. The solid line indicates patients who achieved SVR before hepatectomy and the dashed line represents patients without SVR. SVR, sustained virologic response

During the study period, HCC recurrence developed in 27 (46.6%) and 140 (75.2%) patients in the Pre‐SVR and Non‐SVR group, respectively. The 1‐, 3‐, and 5‐year RFS rates were 86.2%, 62%, and 50.6% in the Pre‐SVR group and 70.2%, 35.6%, and 28.4% in the Non‐SVR group, respectively (*P* < .001; Figure 2A). For recurrent HCC, the proportion of patients with operation selected as the treatment modality was significantly higher in the Pre‐SVR group (14 of 27 patients: 51.9% vs 15 of 140 patients: 10.7%, *P* < .001). The 3‐, 5‐, and 7‐year OS rates were 90.6%, 87.2%, and 82.8% in the Pre‐SVR group and 79%, 61.4%, and 44.2% in the Non‐SVR group, respectively (*P* < .001; Figure 2B). Zero (0%) and five (2.6%) patients died of liver failure in the Pre‐SVR and Non‐SVR group (*P* < .001), respectively.

To evaluate the precise impact of SVR achieved before hepatectomy, 13 confounding factors related to surgical outcomes were adjusted for. In summary, the preoperative liver function indicators, the extent of liver fibrosis, and the AFP level differed significantly between the two groups before PS matching; however, 50 pairs were matched after PS matching, and all of their corresponding variables were balanced (Table 1). Even after adjusting for confounding factors, the patients in the pre‐SVR group had significantly better long‐term surgical outcomes (RFS and OS) than patients in the Non‐SVR group (*P* < .05, Figure 2C,D).

**FIGURE 3:**
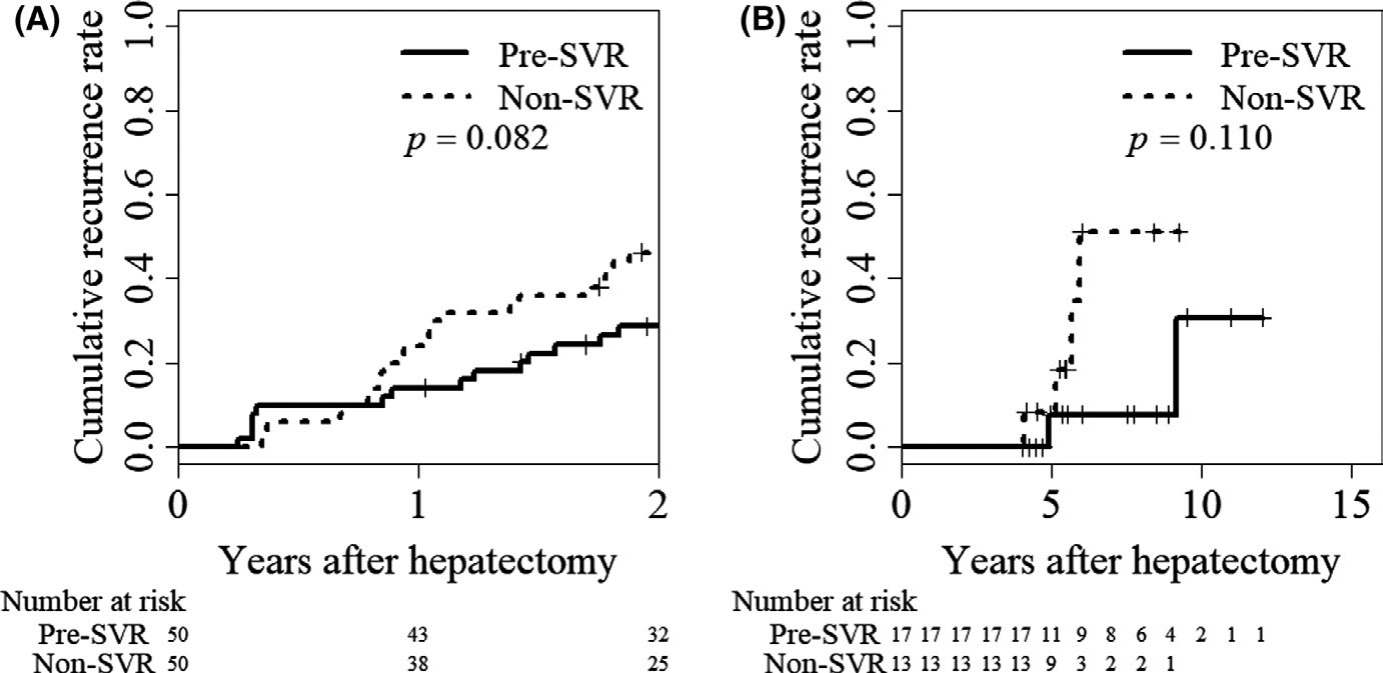
Cumulative incidence rates of HCC recurrence in two different periods according to viral status. Cumulative incidence rates (A) within 2 y after hepatectomy (*P* = .082) and (B) 4 y after hepatectomy (*P* = .11) in the PS‐matched cohort. The Kaplan‐Meier method and log‐rank testing were used to assess the cumulative incidence of HCC recurrence. The solid line indicates patients who achieved SVR before hepatectomy and the dashed line represents patients without SVR. HCC, hepatocellular carcinoma; SVR, sustained virologic response

Furthermore, for the PS‐matched cohort above, we evaluated the potential of SVR before hepatectomy in reducing the HCC recurrence based on two recurrence periods, (early recurrence within 2 years after hepatectomy; and delayed recurrence from 4 years after hepatectomy). In the early recurrence period, the 1‐ and 2‐year cumulative recurrence rates were 14.0% and 28.7% in the SVR group, and 24.0% and 46.3% in the non‐SVR group (*P* = .082; Figure 3A), respectively. In the delayed recurrence period, the 4‐ and 6‐year cumulative recurrence rates were 0% and 7.7% in the SVR group, and 0% and 51.1% in the non‐SVR group (*P* = .11; Figure 3B), respectively. Using multivariate analysis adjusted for factors of early recurrence, a larger size of HCC and achievement of SVR before hepatectomy were identified as independent risk factors for early HCC recurrence (Table S1).

### 3.2 Impact of SVR after hepatectomy on clinical factors and long‐term surgical outcomes

As shown in Figure 1, 166 and 32 patients in the Non‐SVR and Post‐SVR groups, respectively, were analyzed. Patients in the Post‐SVR group were significantly younger (*P* < .001) and had higher albumin levels (*P* = .001) than those in the Non‐SVR group. However, no significant differences occurred in the extent of liver fibrosis between the two groups. Patients in the Post‐SVR group also had significantly smaller tumors (*P* = .006; Table 2).

**TABLE 2:** Clinicopathological factors of the patients with Non‐SVR and Post‐SVR

	Unmatched cohort			IPW‐matched cohort		
	Non‐SVR	Post‐SVR	*P* value	Non‐SVR	Post‐SVR	*P* value
	(N = 166)	(N = 32)		(N = 196)	(N = 154.7)	
Age (y)	72.0 [66, 77]	67.0 [63.8, 70]	**<.001**	71.0 [66, 76]	68 [64, 74]	.104
Male gender, n (%)	117 (70.5)	20 (62.5)	.492	135.5 (69.1)	89.2 (57.6)	.035
Liver function						
Platelet (×10^4^/mL)	12.6 [9.6, 15.4]	13.2 [10.1, 16.4]	.160	12.8 [9.6, 15.3]	13.0 [11, 16]	.312
Albumin (g/dL)	3.7 [3.5, 4]	4.0 [3.8, 4.1]	**.001**	3.7 [3.5, 4]	3.8 [3.6, 4.1]	.281
PT (%)	84.0 [78, 94]	86.0 [79.8, 90.2]	.712	84 [77.0, 94]	83 [75.7, 89]	.699
Liver fibrosis, n (%)						
F0	2 (1.2)	0 (0)	.688	2 (1)	0 (0)	.481
F1	18 (10.8)	3 (9.4)		20.9 (10.6)	17.3 (11.2)	
F2	43 (25.9)	5 (15.6)		48.3 (24.7)	28.8 (18.6)	
F3	34 (20.5)	9 (28.1)		42.7 (21.8)	42.1 (27.2)	
F4	69 (41.6)	15 (46.9)		82.2 (41.9)	66.5 (43)	
Tumor factor						
Maximum tumor Size (cm)	3.0 [2.2, 4]	2.5 [1.7, 3.1]	**.006**	3 [2.1, 4]	3 [2, 4]	.735
Solitary tumor (%)	127 (76.5)	26 (81.2)	.722	152.1 (77.6)	120.3 (77.7)	1
Microvascular invasion (%)	44 (26.5)	4 (12.5)	.142	48.7 (24.9)	28.2 (18.2)	.174
Nodular type (%)	86 (51.8)	17 (53.1)	1	102.5 (52.3)	93.3 (60.3)	.162
Tumor differentiation, poor (%)	27 (16.3)	3 (9.4)	.425	29.9 (15.2)	14.5 (9.4)	.139
AFP (ng/mL)	17.5 [7, 126.5]	10.5 [6.0, 127.5]	.223	17 [7, 119.3]	12 [7.8, 44.1]	.661
Anatomical resection (%)	78 (47)	14 (43.8)	.887	91.2 (46.5)	67.8 (43.8)	.691

Notes

Values are medians (interquartile ranges) or numbers (%). Significant *P*‐values are shown in bold.

Abbreviations: AFP, α‐fetoprotein; IPW, inverse probability weighting; SVR, sustained virological response.

**FIGURE 4:**
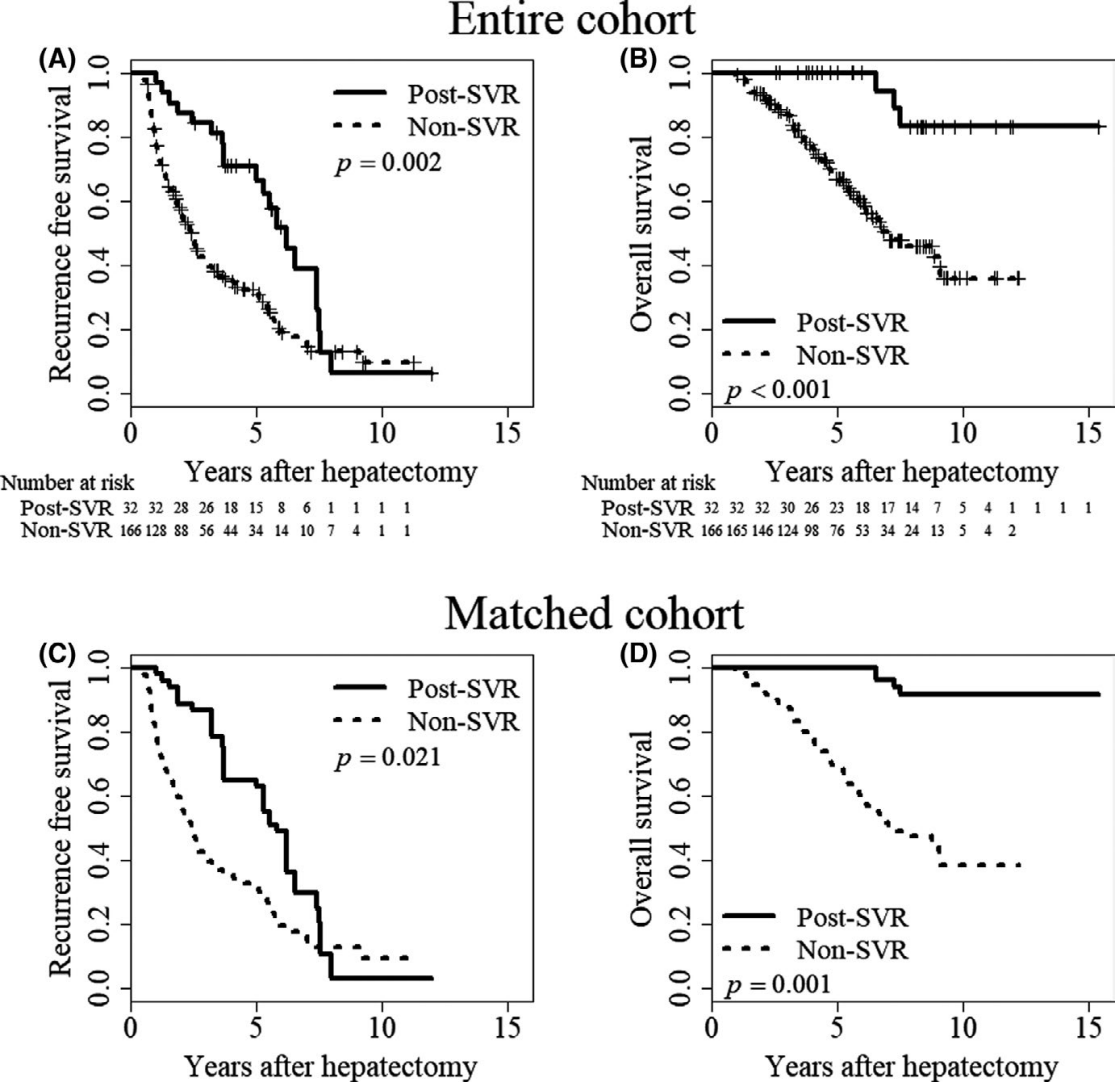
Surgical outcomes of patients in the Non‐SVR group and Post‐SVR group. A, Recurrence‐free survival (*P* = .002) and (B) overall survival (*P* < .001) in the entire cohort. C, Recurrence‐free survival (*P* = .021) and (D) overall survival (*P* = .001) in the inverse probability weighted‐matched cohort. The Kaplan‐Meier method and log‐rank testing were used to assess recurrence‐free survival and overall survival rates. The solid line indicates patients who achieved SVR after hepatectomy and the dashed line represents patients without SVR. SVR, sustained virologic response

During the study period, HCC recurrence developed in 20 (62.5%) and 121 (72.9%) patients in the Post‐SVR and Non‐SVR groups, respectively. Similar to the Pre‐SVR group, the proportion of patients with operation selected as the treatment modality for recurrent HCC was higher in the Post‐SVR group than in the Non‐SVR group (seven of 20 patients: 35% vs 13 of 121 patients: 10.7%, *P* = .0037). Zero (0%) and four patients (2.4%) died of liver failure in the Post‐SVR and Non‐SVR group, respectively. Patients in the Post‐SVR group had significantly better long‐term surgical outcomes (RFS and OS) than those in the Non‐SVR group before (*P* = .002 and *P* < .001) and after (*P* = .021 and *P* = .001) matching for confounding factors (Figure 4). Among the Post‐SVR group, HCC recurrence rates did not differ between patients who received IFN‐based therapy and DAA therapy after hepatectomy (*P* = .37, data not shown).

### 3.3 Influence of SVR on liver fibrosis

**FIGURE 5:**
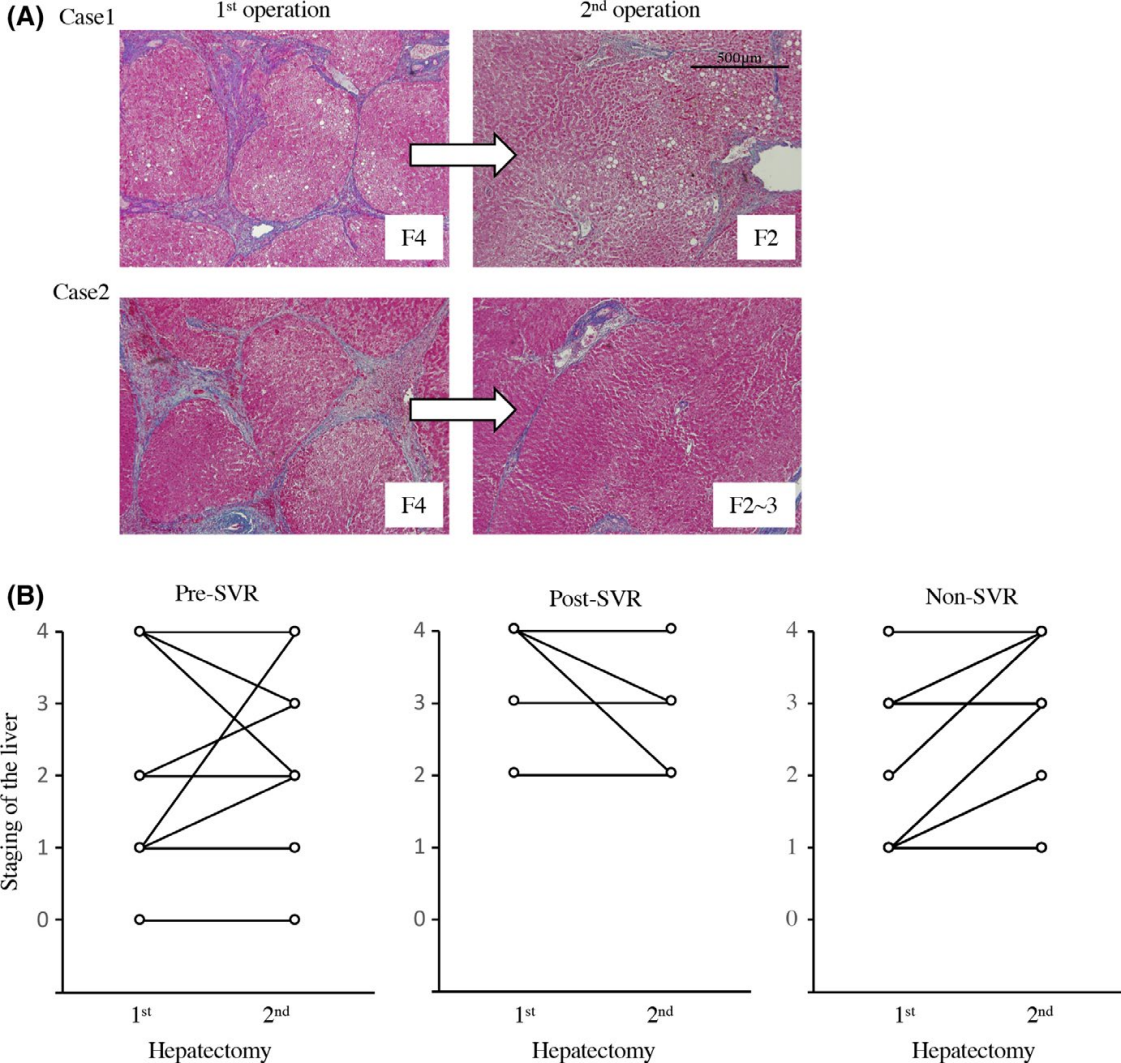
Regression of fibrosis after achievement of SVR. A, Representative data of the change in fibrosis of the resected specimens at first and repeated hepatectomy. Case 1 achieved SVR before hepatectomy, Case 2 achieved SVR after hepatectomy. B, Summary of the change in the liver fibrosis between first and second hepatectomy among patients with preoperative SVR, postoperative SVR, and without SVR. SVR, sustained virologic response

To evaluate whether achievement of SVR could bring about the regression of liver fibrosis, change in liver fibrosis between the first and second hepatectomy were analyzed between three groups (Figure 5). During the study period, 35 patients underwent repeat hepatectomy for recurrent HCC. Figure 5A shows the representative data comparing the immunohistochemistry of non‐cancerous liver parenchyma of the resected specimen between the first and second hepatectomy. Case 1 was a 67‐year‐old man who achieved SVR about 107 months before the first hepatectomy (Pre‐SVR group). The patient underwent a second hepatectomy about 31 months after the first hepatectomy. Regression of liver fibrosis, i.e. from stage 4 to stage 2, was observed. Case 2 was a 63‐year‐old female who achieved SVR 12 months after the initial hepatectomy (Post‐SVR group). The patient underwent a second hepatectomy 80 months after achieving SVR. No change in the degree of liver fibrosis was observed. However, at the third hepatectomy, which was performed 114 months after achieving SVR, regression of liver fibrosis was observed (from stage 4 to stage 2/3). Summarized data are shown in Figure 5B. In summary, two of 13 patients (15.0%) in the Pre‐SVR group and two of five patients (40.0%) in the Post‐SVR group had pathologically improved fibrosis at the second hepatectomy compared to that at the first. Contrariwise, none of the patients in the non‐SVR group showed improved fibrosis (*P* = .038). Moreover, all these patients with improved fibrosis remained alive during the follow‐up period.

## 4 DISCUSSION

In this study, we evaluated the impact of SVR on surgical outcomes for HCC and liver fibrosis. We found that achievement of SVR before and after hepatectomy improved the long‐term surgical outcomes through preventing the intrahepatic recurrence. Regardless of when SVR was achieved (before or after hepatectomy), SVR brought about the regression of liver fibrosis for non‐cancerous liver parenchyma.

Theoretically, achievement of SVR before hepatectomy improves the long‐term surgical outcomes.^5^, ^6^, ^7^, ^8^, ^9^, ^10^, ^11^ However, in almost all reports, the background clinicopathological factors of patients with or without SVR were quite different; that is, patients in the SVR group were younger, had better liver function, less liver fibrosis, and lower levels of AFP at the time of the initial hepatectomy. Therefore, to clarify the impact of achieving SVR before hepatectomy on surgical outcomes, we used PS matching analysis and balanced 13 confounding factors which were chosen based on the previous reports.^13^, ^14^

Even after adjusting for clinicopathological factors, achievement of SVR before hepatectomy was associated with significantly better OS. In this regard, we did not include the details of viral factors, i.e. viral load before antiviral therapy, genotype and the number of mutations within specific regions of the HCV genome and so on, which have been reported to influence efficacy of antiviral treatment. This is because there is minimal credible data that the details of viral factors influence surgical outcomes and few data were available for analysis in this study. Further study with adequate data is warranted for this matter.

The reason for the prolonged OS by preoperative SVR could be explained by two possible mechanisms (suppression of intrahepatic recurrence and preservation of liver functions). For intrahepatic recurrence, some reports supported our result, demonstrating the favorable effect of preoperative SVR on RFS^5^, ^9^, ^24^; however, other reports failed to demonstrate this effect.^6^, ^10^ This discrepancy occurred in part because of the smaller sample sizes compared to the many confounding factors to be adjusted for. To the best of our knowledge, our study is the largest, and a well‐defined multicenter study using PS matching analysis. Therefore, our results strongly implied the possibility that preoperative SVR could improve survival after hepatectomy by preventing recurrence.

In general, postoperative HCC tumor recurrence results from two different processes (the growth of undetectable occult intrahepatic metastasis prior to treatment and the emergence of metachronous multicentric carcinogenesis).^25^ Although assessment of recurrence clonality by genetic/genomic analyses requires the precise differentiation of these recurrence types, these have been reported to depend on the postoperative periods. For example, multicentric carcinogenesis occurs at a constant rate throughout the follow‐up, whereas intrahepatic metastasis mainly occurs in the early postoperative period (generally within 2 years after hepatectomy).^19^, ^26^ On the types of recurrence, we demonstrated the possibility that preoperative SVR could suppress both early and delayed recurrence. For the early recurrence, although the log‐rank RFS analysis curve within 2 years after hepatectomy did not reach a statistically significant level, the multivariate analysis showed that achievement of SVR before hepatectomy was an independent predictive factor for suppression of early recurrence. This is noteworthy, because this implies the possibility that achievement of SVR before hepatectomy could suppress not only multicentric carcinogenesis but also intrahepatic metastasis. Previously, several studies have reported that preoperative SVR could suppress multicentric carcinogenesis^5^, ^9^, ^11^, ^13^, ^24^; however, there was only one report that indicated the suppressive impact of preoperative SVR on early recurrence after hepatectomy.^27^ Furthermore, when the recurrence‐free curves reported in a previous article by Kunimoto et al were compared in detail between the PS‐matched groups, the curves for the Pre‐SVR group represented apparently better outcomes than that for the Non‐SVR group in both the early and delayed periods.^24^ From other previous reports, although the background factors were not adjusted for, a similar trend was found.^8^, ^10^, ^24^, ^28^ Therefore, we believe the possibility that preoperative SVR could reduce intrahepatic metastasis. Possible molecular mechanisms of the suppressive effect of SVR on HCC recurrence could include the future regression of liver fibrosis, improvement in immune surveillance against carcinogenesis, or alternation of gene expression due to the resolution of inflammation.^29^, ^30^, ^31^ Further in vitro and in vivo research is warranted for elucidating the mechanisms of preoperative SVR to suppress both early and delayed HCC recurrence after hepatectomy.

For the liver function, hepatic decompensation is the major factor of death in HCV‐infected cirrhotic patients who undergo curative treatment for HCC. In this study, no patient died of liver failure in the SVR group while five in the Non‐SVR group died. Moreover, consistent with previous reports, a higher proportion of patients for whom operation was selected as the treatment modality for the recurrent HCC occurred in the Pre‐SVR group than the Non‐SVR group.^6^, ^9^, ^10^, ^11^ Therefore, achievement of SVR before hepatectomy could improve OS by preventing the worsening of the liver function and by providing the opportunity for patients to undergo curative resection for recurrent HCC.

Secondly, achievement of SVR after hepatectomy improved OS and RFS. Compared to the many previous reports that evaluated the effect of preoperative SVR, only a few evaluated the effect of SVR after hepatectomy.^11^, ^12^, ^13^ With regard to the OS, almost all previous reports demonstrated the favorable effect of SVR after curative treatment; however, it still has been debated whether achieving SVR after curative treatment for HCC could suppress recurrence.^12^, ^32^, ^33^ Because our study was still too small to adjust for the confounding factors, we performed inverse probability of treatment weighting analysis to evaluate precisely the impact of postoperative SVR on surgical outcomes. Thus, we demonstrated a significantly favorable effect of postoperative SVR on OS and RFS. The same results were obtained using PS matching analysis (data not shown). In addition to the improvement of liver function, various mechanisms of IFN, e.g. the anti‐tumor effect and systemic immunity effect, and so on could be related to this suppressive effect.^29^, ^31^, ^34^ Recently, there is still a controversy regarding suppressive effect of DAA for HCC recurrence after curative treatment.^35^, ^36^ Although our study had a small sample size, there was no difference about HCC recurrence rates between patients who received IFN‐based therapy and DAA therapy after curative resection of HCC. Further study with larger sample size and longer observational period is needed to confirm these results.

Finally, regardless of the period, SVR brought about the regression of the fibrosis of non‐cancerous liver parenchyma. Fibrosis of the liver has been reported as an independent prognostic factor even after curative resection of HCV‐related HCC.^6^, ^7^ Therefore, there is a vital need to clarify the relationship between achieving SVR and change in liver fibrosis. Previous studies have elucidated that viral elimination can bring about regression of fibrosis in non‐cancerous patients with HCV‐compensated cirrhosis.^37^, ^38^, ^39^ However, only two studies reported the possibility that achieving SVR could be associated with regression of liver fibrosis in patients with HCV‐related HCC using liver biopsy sections.^8^, ^40^ However, considering the variability in the distribution of fibrosis within the liver, using the whole resected specimen obtained during the operation is theoretically ideal to precisely evaluate for change in liver fibrosis.^41^ To the best of our knowledge, this is the first study to evaluate the relationship between SVR and regression of liver fibrosis in patients with HCV‐related HCC, using whole resected liver specimens. In this study, we elucidated that only patients with SVR had significantly improved liver fibrosis at the repeated hepatectomy compared to at the first hepatectomy (about 20%). The regression rate varies between studies previously reported.^8^, ^38^, ^39^, ^40^ Many factors are still needed to be uncovered, including the subgroup of patients in whom regression of fibrosis is possible and how to determine whether such regression has occurred (e.g. hepatic venous pressure gradient, liver stiffness, and absence or presence of HCC).^40^, ^42^, ^43^

In conclusion, our results provided strong evidence that achievement of SVR, regardless of timing, is essential for improving surgical outcomes in patients with HCV‐related HCC. Currently, with the dramatic change in antiviral therapy by the development of IFN‐Free DAAs, more patients with HCV‐related HCC will achieve SVR before or after hepatectomy. It is premature to evaluate the influence of SVR by DAA treatment on long‐term surgical outcomes after curative resection of HCC; therefore, this study can be a benchmark for a new era of antiviral therapy to assess the importance of SVR on surgical outcomes of HCV‐related HCC.

## DISCLOSURE

Funding: This research was partially supported by the Ministry of Education, Science, Sports, and Culture, Grant‐in‐Aid for Scientific Research.

Conflict of Interest: The authors declare that they have no conflict of interest.

Author contributions: Conception and design; Masao Nakajima, Shogo Kobayashi, Hiroshi Wada and Hiroaki Nagano. Acquisition of the data: Masao Nakajima, Shogo Kobayashi, Hiroshi Wada, Akira Tomokuni, Hidenori Takahashi, Takehiro Noda, Hiroto Matsui, Satoshi Matsukuma, Yoshitaro Shindo, Yukio Tokumitsu, Nobuaki Suzuki, Shigeru Takeda, Hidetoshi Eguchi and Hiroaki Nagano. Analysis and interpretation of the data: Masao Nakajima, Shogo Kobayashi, Hiroshi Wada, Yuki Nakagami, Yoshinobu Hoshii, Katsuyoshi Ito and Hiroaki Nagano. Drafting of the manuscript: Masao Nakajima, Shogo Kobayashi, Hiroshi Wada, Yuki Nakagami, Masahiro Tanabe and Hiroaki Nagano. Statistical analysis: Masao Nakajima, Shogo Kobayashi, Hiroshi Wada and Yuki Nakagami. Study supervision: Hidetoshi Eguchi and Hiroaki Nagano. All the authors gave the final approval for publication, and agreed to be accountable for all aspects of the work.

## ETHICAL STATEMENTS

This study was approved by the Human Ethics Committee in Yamaguchi University (approval no. H30‐046) and all patients provided written informed consent.

## Supporting information

Table S1
