# Supplementary material for: Viral elimination is essential for improving surgical outcomes of hepatitis C virus‐related hepatocellular carcinoma: Multicenter retrospective analysis
Source: Ann Gastroenterol Surg. 2020 Jul 23;4(6):710–20. doi: 10.1002/ags3.12377 (PMC7726693; doi:10.1002/ags3.12377)
Supplement: Supplementary file 1 — Table S1 [file AGS3-4-710-s001.docx]

| **Table S1. Multivariate analysis for early recurrence in the propensity matched cohort** | | | |
| --- | --- | --- | --- |
| Factors | OR | 95% CI | P value |
| Maximum tumor Size (cm) | 1.16 | 1.00-1.34 | **0.032** |
| Solitary tumor | 0.55 | 0.29-103 | 0.063 |
| Microvascular invasion | 1.25 | 0.66-2.36 | 0.49 |
| Nodular type | 0.68 | 1.16 | 0.15 |
| Tumor differentiation, poor | 1.84 | 0.95-3.7 | 0.07 |
| AFP (ng/ml) | 0.99 | 0.99-1.00 | 0.92 |
| Antomical resection | 0.76 | 0.34-0.43 | 0.33 |
| achievement of preoperative SVR | 0.44 | 0.22-087 | **0.014** |
| Significent P-values are shown in bold.  Abbreviations: SVR, Sustained virological response; AFP, α-fetoprotein | | | |
